# Studying the relationship between intelligence quotient and schizophrenia polygenic scores in a family design with first-episode psychosis population

**DOI:** 10.1192/j.eurpsy.2024.24

**Published:** 2024-03-11

**Authors:** Nancy Murillo-García, Sergi Papiol, Luis Manuel Fernández-Cacho, Mar Fatjó-Vilas, Rosa Ayesa-Arriola

**Affiliations:** 1 Research Group on Mental Illnesses, Valdecilla Biomedical Research (IDIVAL), Santander, Spain; 2 Department of Molecular Biology, School of Medicine, University of Cantabria, Santander, Spain; 3 Department of Falkai, Max Planck Institute of Psychiatry, Munich, Germany; 4 Biomedical Research Networking Center for Mental Health (CIBERSAM), Health Institute Carlos III, Madrid, Spain; 5 Department of Radiology, Marqués de Valdecilla University Hospital, Santander, Spain; 6 Faculty of Nursing, University of Cantabria, Santander, Spain; 7 FIDMAG Sisters Hospitallers Research Foundation, Barcelona, Spain; 8 Departament de Biologia Evolutiva, Ecologia i Ciències Ambientals, Facultat de Biologia, Universitat de Barcelona, Barcelona, Spain

**Keywords:** family, first-episode psychosis, intelligence quotient, polygenic risk score, polygenic scores

## Abstract

**Background:**

The intelligence quotient (IQ) of patients with first-episode psychosis (FEP) and their unaffected relatives may be related to the genetic burden of schizophrenia (SCZ). The polygenic score approach can be useful for testing this question.

**Aim:**

To assess the contribution of the polygenic risk scores for SCZ (PGS-SCZ) and polygenic scores for IQ (PGS-IQ) to the individual IQ and its difference from the mean IQ of the family (named family-IQ) through a family-based design in an FEP sample.

**Methods:**

The PAFIP-FAMILIES sample (Spain) consists of 122 FEP patients, 131 parents, 94 siblings, and 176 controls. They all completed the WAIS Vocabulary subtest for IQ estimation and provided a DNA sample. We calculated PGS-SCZ and PGS-IQ using the continuous shrinkage method. To account for relatedness in our sample, we performed linear mixed models. We controlled for covariates potentially related to IQ, including age, years of education, sex, and ancestry principal components.

**Results:**

FEP patients significantly deviated from their family-IQ. FEP patients had higher PGS-SCZ than other groups, whereas the relatives had intermediate scores between patients and controls. PGS-IQ did not differ between groups. PGS-SCZ significantly predicted the deviation from family-IQ, whereas PGS-IQ significantly predicted individual IQ.

**Conclusions:**

PGS-SCZ discriminated between different levels of genetic risk for the disorder and was specifically related to patients’ lower IQ in relation to family-IQ. The genetic background of the disorder may affect neurocognition through complex pathological processes interacting with environmental factors that prevent the individual from reaching their familial cognitive potential.

## Introduction

Intelligence quotient (IQ) is a quantitative estimate of an individual’s general cognitive ability [1]. Patients experiencing a first-episode psychosis (FEP) tend to have lower IQs than healthy controls [2,3]. It has also been described that these IQ deficits precede the onset of psychosis, probably due to neurodevelopmental impairments [4,5]. While cognitive abilities aggregate in families, FEP patients tend to perform worse on cognitive tasks than their first-degree relatives, indicating a deviation from familial cognitive aptitude [6–10]. Accordingly, IQ and specific neuropsychological functions have been largely investigated as endophenotypic traits of psychosis that may enhance preventive measures and early intervention [11–14].

Both IQ and psychosis are highly heritable, with heritability estimates ranging from 40% to 70% [15,16] and 60% to 80% [17,18], respectively. The polygenic score (PGS) method is useful for estimating an individual’s genetic make-up for such complex phenotypes [19,20]. On the one hand, it is possible to calculate PGS-IQ based on the results of large-scale genome-wide studies that have characterised the genetic architecture of intelligence [21]. PGS-IQ is strongly correlated with crystallised intelligence and accounts for up to 5.1% of the variance in general cognitive ability [22]. On the other hand, polygenic risk scores for schizophrenia (PGS-SCZ) can be calculated leveraging the results of genome-wide studies on this disorder [23,24]. PGS-SCZ explain between 2.4% and 7.3% of the variance in SCZ on the liability scale [23,24] and is increased in FEP patients compared to controls [25,26]. There may be a certain degree of association between these two PGSs, given that numerous genetic variants have been identified as contributing factors to intelligence and SCZ [27,28]. Similarly, PGS discriminating SCZ from bipolar disorder was found to be specifically related to intelligence [29].

We hypothesised that i) FEP patients would have higher PGS-SCZ and lower PGS-IQ than first-degree relatives and healthy controls, and ii) PGS-SCZ would be negatively associated with IQ and the patient’s IQ deviation from the mean score of their family (named family-IQ), suggesting that genetic predisposition to SCZ is related to worse general cognitive ability. We also expected a positive association of PGS-IQ with IQ.

Our primary aim was to test whether the genetic risk for SCZ, as determined by PGS-SCZ, might be associated with IQ and contributed to patient-specific differences from their family-IQ in a sample of FEP patients, their first-degree relatives, and healthy controls. Second, we also aimed to examine to what extent PGS-IQ predicts intelligence and deviation from family-IQ.

## Methods

### Sample

Participants were drawn from PAFIP-FAMILIES, a family-based study carried out in Cantabria, Spain, from January 2018 to March 2021, funded by the ISCIII (FIS PI17/00221). All participants were of European ancestry. We recruited first-degree relatives of a cohort of FEP patients previously enrolled in the Cantabria Program for Early Intervention in Psychosis (PAFIP) [30,31]. The local institutional review committee (CEIm Cantabria) approved both projects (PAFIP and PAFIP-FAMILIES) under international research ethics standards and all participants gave their written informed consent. The initial sample consisted of 133 FEP patients, 146 parents, 98 siblings, and 202 controls [32].

#### FEP patients

The PAFIP program was carried out at the University Hospital Marqués de Valdecilla (Santander, Spain) from 2001 to 2018. FEP patients were referred from the inpatient unit, outreach mental health services, and healthcare centres in the region. Inclusion criteria were as follows: 1) 15–60 years of age; 2) living within the recruitment area; 3) experiencing an FEP; 4) no prior treatment with antipsychotic medication or if previously treated, a total lifetime of antipsychotic treatment of <6 weeks; and 5) criteria for brief psychotic disorder, schizophreniform disorder, SCZ, or not otherwise specified psychosis according to the Diagnostic and Statistical Manual of Mental Disorders, fourth edition (DSM-IV). The exclusion criteria included meeting the DSM-IV criteria for drug or alcohol dependence, having an intellectual disability, and having a history of neurological disease or head injury.

#### First-degree relatives

We contacted the parents and siblings of the eligible patients (those with neuropsychological data and DNA samples) and invited them to participate in the study. Inclusion criteria were as follows: 1) age over 15 years, 2) good domain of the Spanish language, and 3) ability to give informed consent in writing. Exclusion criteria included a history of psychiatric diagnosis related to psychotic illness spectrum, organic brain pathology, and intellectual disability or substance use disorders according to DSM-V criteria.

#### Controls

Controls were retrieved from the PAFIP program, which recruited healthy individuals through advertisements from the local community. They met the same inclusion and exclusion criteria as first-degree relatives. The psychiatric history of controls and relatives was screened by the abbreviated version of the Comprehensive Assessment of Symptoms and History [33], a semi-structured psychiatric interview that inquires about the presence of clinical symptoms for mania, depression, and positive, disorganised, and negative dimensions of psychosis.

### Phenotypic data

#### Sociodemographic data

We recorded the sex, age, and completed years of formal education of all participants. Cannabis consumption was recorded for FEP patients, siblings, and controls.

#### Clinical data

We obtained clinical data from patients at baseline through medical records and interviews. The age at psychosis onset was defined as the age when the emergence of the first continuous psychotic symptom occurred. Duration of untreated illness was defined as the time from the first nonspecific symptom related to psychosis. Duration of untreated psychosis was established as the time from the first continuous psychotic symptom to initiation of antipsychotic drug treatment. Patients were randomly assigned to treatment with olanzapine, risperidone, or haloperidol [34]. Positive symptoms were assessed by the Scale for the Assessment of Positive Symptoms [35], and negative symptoms by the Scale for the Assessment of Negative Symptoms [36]. Functioning was rated by the Global Assessment of Functioning [37]. Diagnoses were confirmed through the Structured Clinical Interview for DSM-IV (SCID-I) conducted by an experienced psychiatrist within 6 months of the baseline visit.

#### Estimation of IQ

Expert neuropsychologists administered the WAIS-III Vocabulary subtest [1] to estimate the IQ of all participants. This subtest has adequate properties as a proxy measure for crystallised intelligence in the general population and FEP [38]. Crystallised intelligence is defined as knowledge acquired throughout life, including vocabulary, general information, culture, and specific skills [39]. It represents the stored information and strategies that individuals draw on to solve common problems [40]. Crystallised intelligence is more stable than fluid intelligence [41]; thus, the Vocabulary subtest would enable the estimation of cognitive abilities before the onset of psychosis in the FEP sample. This subtest is associated with educational attainment and the linguistic knowledge of one’s native language [41]. We have previously used Vocabulary as a proxy measure for premorbid intelligence, showing utility in studying the IQ of FEP patients [42].

**Figure 1.**
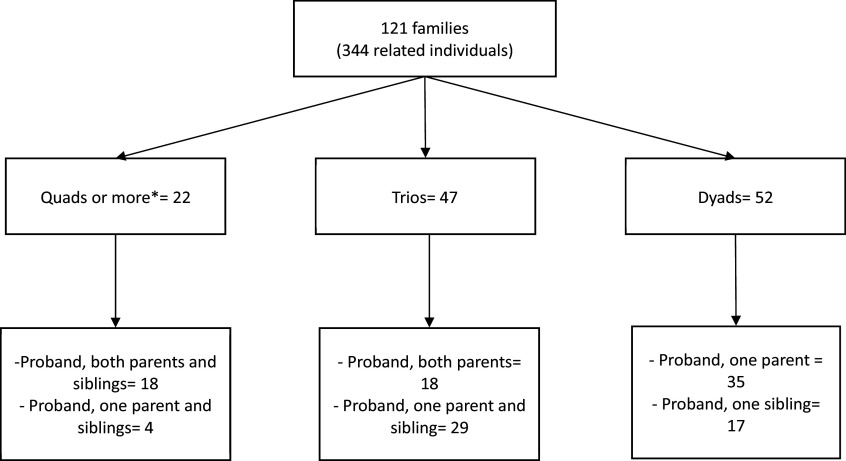
Conformation of the families participating in this study. *Note*: Each family was formed by a FEP patient and at least one first-degree relative, either a parent or sibling. All participants completed the same neuropsychological battery and provided a DNA sample that allowed the calculation of polygenic scores. *There was one family with nine members, one with six members, and five with five members.

To estimate a proxy of the potential IQ of FEP patients, we calculated a “family-IQ” for each family. This score represents the mean IQ of all family members, including the FEP patient themself. We included patients in the estimation because 42% of our families consisted of only the proband and one other member (see Figure 1). See the details of family-IQ estimated from unaffected relatives only in the Supplementary Material.

Deviation from family-IQ was determined by calculating the difference between the individual and family scores. Positive deviations indicate that an individual’s IQ is above their family-IQ, while negative deviations indicate that it is below their family-IQ.

### Genotyping and PGSs estimation

DNA was extracted from venous blood samples at baseline. Samples and data from patients included in this study were provided by the Biobank Valdecilla (PT20/00067), integrated into the Spanish Biobank Network and they were processed following standard operating procedures with the appropriate approval of the Ethical and Scientific Committees. The genotyping was performed at the Centro Nacional de Genotipado (Human Genotyping laboratory, CeGen) using the Global Screening Array v.3.0 panel (Illumina).

The quality control process was performed using PLINK 1.9. Single-nucleotide polymorphisms (SNPs) with a minor allele frequency of less than 0.01, missing data exceeding 0.02, or exhibiting deviation from Hardy–Weinberg equilibrium were removed. Participants were excluded if there were discrepancies in sex information or detected heterozygosity. A set of SNPs meeting high-quality criteria (HWE *p* > 0.001, MAF > 0.01) and subjected to linkage disequilibrium pruning was employed to assess relatedness. We confirmed the participants’ recorded relationships, in which PI-HAT values around 0.50 were considered to indicate first-degree relatives. Ancestry outliers were identified through principal component analysis based on 1000 Genomes Project European reference populations and subsequently removed (see Supplementary Figure S1). The final dataset comprised 525 participants and 492,348 SNPs. Genetic imputation was carried out in the Michigan Imputation Server using Minimac4 and individuals from the Haplotype Reference Consortium (HRC; Version r1.1) as the reference dataset. Genetic variants with MAF > 0.01 were kept. After imputation, 6,910,431 SNPs were available for downstream analyses.

We calculated PGS for each participant using the latest publicly available summary statistics for SCZ [23] and IQ [21] by the method of polygenic continuous shrinkage (PGS-CS) [43]. PGS-CS shrinks the effect sizes towards the population mean, thereby attenuating the influence of variants with unstable or exaggerated effects. This regularisation technique provides more reliable and interpretable PGS estimates, enhancing their predictive power and generalizability across different populations or cohorts. PGS was then calculated in PLINK 1.9 using imputed dosage data in this cohort.

After obtaining the PGS in our sample, we corrected it by their first five ancestry principal components. The aim was to control for their possible influence on our results. We regressed the effect of the principal components on the PGS using a linear model. Finally, we kept the residuals as the corrected PGS and standardised them.

### Statistical analysis

We performed statistical analysis in R [44]. To take into account that our sample was related, we carried out linear mixed models (LMMs) using the “lme4” package.
(1)

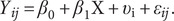

In Equation 1), 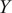 represents the dependent variable. The subscripts 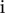 and 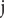 on the 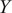 indicate that each observation 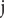 is nested within cluster 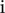 , in this case, the family. 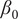 is the overall intercept. 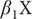 represents the vector of fixed effects. 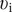 is the random effect of family code. 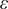 is the error of the model. We adjusted the *p*-values by false discovery rate (FDR) and considered those equal to or less than 0.05 as significant.

Between-group comparisons were performed using separate LMMs, one for each dependent variable (IQ, deviation from family-IQ, PGS-SCZ, PGS-IQ, and sociodemographic) according to Equation (1). These models included the grouping variable as a fixed effect (FEP patient, sibling, parent, or control) and the family code as a random effect. We covariated IQ comparisons by sex, age, and years of education. Post hoc comparisons were conducted with Bonferroni correction and effect sizes were estimated using beta standardised coefficients.

Then, we performed the main analyses, consisting of four LMMs according to Equation 1), which were fitted to families without controls. All four models included the same covariates (sex, age, and years of education) and random effect (family code). The first and second models tested the predictive effect of PGS-SCZ on IQ and deviation from family-IQ, respectively. The third and fourth models tested the predictive effect of PGS-IQ on IQ and deviation from family-IQ, respectively.

We tested the potential effect of antipsychotic medication (chlorpromazine-equivalent dose at baseline) on patients’ IQ and found no significant results (*p* = 0.585). Therefore, the antipsychotic variable was excluded from the main analyses.

## Results

### Descriptive statistics and between-group comparisons

Of all subjects with PGS estimates, five were removed from the LMM analyses because they could not be nested within families (e.g., a dyad whose family member was removed in QC becomes incomplete). The final sample consisted of 344 relatives and 176 controls. Figure 1 displays the distribution of the 121 families included in the LMMs.

There was a higher proportion of men in the FEP and control groups compared to siblings and parents (*p* < 0.001). FEP patients were significantly younger than all other groups and had higher rates of cannabis use than controls and siblings (*p* < 0.050). Siblings were significantly older than controls and had completed more years of education than the other participants had (*p* < 0.001).

**Figure 2.**
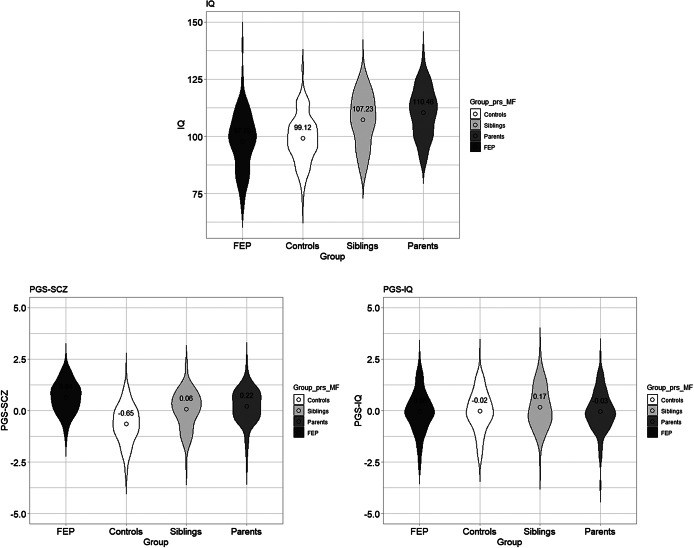
Violin plots of IQ, PGS-SCZ, and PGS-IQ according to the group of participants. *Note*: The IQs shown in the first plot are without corrections for age and years of education. After introducing the former covariates, parents had higher IQs than FEP patients (*p* = 0.024) and controls (*p* = 0.018). Regarding PGS-SCZ, FEP patients had higher scores than all other groups (*p* < 0.001). No significant differences were found for PGS-IQ.

Table 1 shows post hoc comparisons between groups. After correcting for covariates, parents had significantly higher IQs than patients (*p* = 0.024) and controls (*p* = 0.018). FEP patients deviated more from family-IQ (*p* < 0.001) than their relatives. The FEP patients had significantly higher PGS-SCZ than all other groups (*p* < 0.001), and their parents had significantly higher PGS-SCZ than controls (*p* = 0.023) (Figure 2). PGS-IQ was not different between groups.

**Table 1.** Between-group comparisons using linear mixed model analysis

	FEP patients (n = 121)			Parents (n = 131)			Siblings (n = 92)			Healthy controls (n = 176)			*F*	*p*	Post hoc comparisons
	Mean	SD	Effect size (β Std)	Mean	SD	Effect size (β Std)	Mean	SD	Effect size (β Std)	Mean	SD	Effect size (β Std)			
IQ	97.44	13.04	−0.06	110.46	10.66	0.39	107.23	11.30	0.26	99.12	10.55	0.00*	49.35	<0.001	P > FEP, HC; S > FEP, HC
IQ with covariates*	99.02	SE= 1.10	−0.02	107.66	SE=1.40	0.22	100.26	SE=1.08	0.13	100.34	SE=0.89	0.00^a^	4.74	0.003	P > FEP (*p* = 0.024), HC (*p* = 0.018)
Deviation from family–IQ	−6.84	8.74	−0.46	4.83	7.78	0.12	2.48	7.95	0.00^b^	NA	NA	NA	69.80	<0.001	FEP < P, S
PGS–SCZ	0.79	0.08	0.50	−0.08	0.08	0.12	−0.20	0.09	0.06	−0.38	0.07	0.00^a^	45.83	<0.001	FEP > HC, S, P HC < P (0.023)
PGS–IQ	−0.12	0.09	−0.04	0.03	0.09	0.02	0.14	0.011	0.06	−0.01	0.08	0.00^a^	1.21	0.305	NS
*Sociodemographics*															
Sex (male %)	73 (60.3%)		−	49 (37.4%)		−	32 (34.8%)		−	106 (60.23%)		−	χ= 29.36	<0.001	FEP> S, P; HC> S, P
Age	26.99	8.61	−0.08	62.06	7.72	0.88	40.72	13.22	0.18	30.20	8.29	0.00^a^	386.72	<0.001	FEP < HC (*p* = 0.026), S, P; P > all, S>HC
Years of education	10.67	3.47	−0.04	10.20	3.50	−0.11	12.65	3.66	0.19	11.01	2.69	0.00^a^	11.24	<0.001	S > all
Cannabis consumption (yes%)	55 (45.5%)		−	NA		−	5 (5.32%)		−	21 (11.93%)		−	*χ* = 74.12	<0.001	FEP > HC, S
*Clinical at baseline*															
Diagnosis (schizophrenia %)	55 (45.08%)														
Age at psychosis onset	26.15	8.42													
SAPS	14.55	4.86													
SANS	6.59	6.28													
DUI	20.09	32.55													
DUP	12.94	29.15													
GAF	51.89	30.28													

Abbreviations: DUI, duration of untreated illness; DUP, duration of untreated psychosis; GAF, global assessment of functioning; IQ, intelligence quotient; NA, not available; SANS, scale for the assessment of negative symptoms, SAPS, scale for the assessment of positive symptoms.

*Notes:* All post hoc comparisons were Bonferroni corrected and significant at *p* < 0.001 except when indicated. *IQ was covariated with age and years of education.

a Controls were used as the reference category in the models (intercept). Therefore, the effect sizes of the other groups represent their differences from the controls.

b Siblings were used as the reference category in the models (intercept).

### Predictive effect of the PGSs on IQ and deviation from family-IQ

PGS-SCZ was not associated with IQ (*β* = −0.08, *SE* = 0.04, *p* = 0.53, *pFDR* = 0.63). However, PGS-SCZ significantly predicted IQ deviation from family-IQ (*β* = −0.17, *SE* = 0.05, *pFDR* = 0.003) (see the results detailed in Table 2).

**Table 2.** The predictive effect of PGS-SCZ on IQ and deviation from family-IQ using linear mixed models

Model 1: IQ as dependent variable			
Fixed effects	Beta coefficient standardized (SE)	*T*	*FDRp*
PGS–SCZ	−0.08 (0.04)	–1.940	0.063
Years of education	0.45 (0.04)	8.570	<0.001
Age	0.39 (0.05)	10.230	<0.001
Sex	0.01 (0.04)	0.415	0.678

Model 2: Deviation from family-IQ as dependent variable			
Fixed effects	Beta coefficient standardized (SE)	*T*	*FDRp*
PGS–SCZ	−0.17 (0.04)	–3.547	<0.001
Years of education	0.16 (0.04)	3.461	<0.001
Age	0.40 (0.04)	8.089	<0.001
Sex	0.07 (0.04)	1.502	0.134

Overall model 1: *Wald* = 194.86, *p* < 0.001, *R^2^* = 0.46.

Overall model 2: *Wald* = 115.98, *p* < 0.001, *R^2^* = 0.26.

PGS-IQ significantly predicted the individual IQ (*β* = 0.13, *SE* = 0.04, *pFDR* = 0.003) but showed a trend towards significance in predicting the deviation from family-IQ (*β* = 0.08, *SE* = 0.04, *pFDR* = 0.073) (see the results detailed in Table 3).

**Table 3.** The predictive effect of PGS-IQ on IQ and deviation from family-IQ using linear mixed models

Model 3: IQ as dependent variable			
Fixed effects	Beta coefficient standardized (SE)	*T*	*FDRp*
PGS–IQ	0.13 (0.04)	3.089	0.003
Years of education	0.34 (0.03)	8.993	<0.001
Age	0.47 (0.04)	11.237	<0.001
Sex	0.01 (0.04)	0.242	0.809

Model 4: Deviation from family-IQ as dependent variable			
Fixed effects	Beta coefficient standardized (SE)	*T*	*FDRp*
PGS–IQ	0.08 (0.04)	1.799	0.073
Years of education	0.18 (0.04)	3.738	<0.001
Age	0.44 (0.04)	9.238	<0.001
Sex	0.06 (0.04)	1.301	0.194

Overall model 1: *Wald* = 204.07, *p* < 0.001, *R^2^* = 0.46.

Overall model 2: *Wald* = 104.02, *p* < 0.001, *R* ^2^ = 0.24.

## Discussion

Through a family-based design, we add data on the association of the polygenic background of SCZ and IQ with general cognitive performance. We report, as expected, that PGS-SCZ is increased in FEP patients as compared to their relatives and controls. Our data also show that PGS-SCZ significantly predicts the individual’s deviation from the mean IQ of their relatives, whereas PGS-IQ is more predictive of the individual’s IQ.

### Between-group differences in IQ, PGS-SCZ, and PGS-IQ

FEP patients had higher PGS-SCZ than other groups, with first-degree relatives having intermediate scores. This supports the efficacy of the PGS method in discerning varying levels of genetic predisposition to psychosis. While previous research indicates that PGS-SCZ can differentiate between FEP patients and controls [25,26], our findings suggest that it can also detect genetic risk variation within families. Although FEP patients showed PGS-IQ similar to other groups, their IQ scores were lower, suggesting unachieved cognitive potential. In addition, FEP patients showed a negative deviation from their family-IQ of 6.84 points on average. This is consistent with previous research describing a strong correlation between deviation from family cognitive ability and risk of SCZ [10]. Such deviation is aligned with the well-reported cognitive impairments associated with SCZ [6], bringing at the same time new questions about the aetiological mechanisms underlying the intra-family differences. Thus, deviation from familial aptitude emerges as an important marker of neurodevelopmental processes predisposing to psychosis [10].

We found that unaffected siblings have a lower PGS-SCZ than the proband, implying a slightly reduced genetic predisposition to SCZ. Siblings had similar IQs to controls, and their performance aligned with their family cognitive profile. Previous research consistently shows that siblings tend to perform better than the proband in cognitive domains such as executive functions and memory [6,32,45–47]. Siblings had higher educational attainment and lower cannabis use rates (Table 1), which may be protective factors that increase cognitive reserve against psychosis [48,49].

Parents in our sample were found to have higher IQs than the other participants, including the healthy controls. This finding contrasts with previous evidence showing IQ deficits among first-degree relatives of FEP patients [6,7,9,50,51]. The discrepancy in results may be related to the neuropsychological measure used in our study. We estimated crystallised intelligence, which tends to increase with age [52] and is strongly influenced by education [53]. As parents in our sample are the oldest, age may have contributed to their IQ advantage.

### Relationship between PGS-SCZ and deviation from family-IQ

Our research shows that PGS-SCZ can predict deviation from family-IQ, but it does not have any direct relationship with IQ. These findings converge with some previous studies showing no connection between genetic risk of SCZ and intelligence [54,55]. However, others have reported a direct correlation between higher PGS-SCZ and low intelligence in individuals at high risk of psychosis [56], with SCZ [29], and in controls [57,58]. Conflicting findings in the literature may be due to differences in neuropsychological measures and sample variation. An alternative explanation is that genetic risk for SCZ may influence longitudinal intellectual trajectories rather than cross-sectional IQ scores. Although the literature on FEP is limited, some insights can be drawn from studies of the general population. Germine et al. [59] described that PGS-SCZ was associated with reduced speed of emotion identification and verbal reasoning in childhood. McIntosh et al. [57] found that high PGS-SCZ was associated with greater cognitive decline. Therefore, this evidence suggests that genetic liability for SCZ may be related to specific cognitive domains at key life stages. These trajectories need to be explored in the FEP population, as long-term factors such as antipsychotic medication or disease progression may influence their cognitive outcomes.

Concerning intellectual family deviation, our findings indicate that an increase of one standard deviation in PGS-SCZ may lead to roughly 0.17 standard deviations of negative deviation from family-IQ. Following Kendler et al. [10], we interpret that the genetic liability for SCZ indirectly influences intelligence by disrupting neurodevelopment and preventing the achievement of cognitive potential. In this regard, it could be suggested that increased genetic susceptibility to SCZ in FEP patients may shape developmental trajectories and/or make individuals more sensitive to environmental insults [60,61], leading to the onset of psychosis. This interpretation is based on existing evidence of a common genetic susceptibility between SCZ and neurodevelopmental disorders [62,63], which, when combined with environmental risk factors [60,64], can increase the likelihood of impaired cognitive development from an early age.

### Relationship between PGS-IQ and IQ

We confirmed a strong association between PGS-IQ and IQ. This association has been previously reported in the general population [19,22], and our study replicates it in the FEP population [25,65]. As expected, polymorphic genetic factors explain a small percentage of the variance in IQ, suggesting that there is a very large amount of variability associated not only with other sources of genomic variability but also with environmental factors.

As PGS-IQ showed a trend towards predicting deviation from family-IQ (*p* = 0.073), the evidence for this relationship remains unclear. Deviation from family cognition may not solely reflect the risk of SCZ. It is also possible that a lower genetic predisposition to intelligence contributes to this deviation. Further research on IQ in FEP, particularly investigating indirect parental genetic effects, could provide more clarity [66,67]. Research has shown a robust effect of genetic nurture on education, influenced by parental education and socioeconomic status [68,69]. This pathway could be homologous to IQ, although this needs to be verified in future studies.

#### Strengths and limitations

The strength of this study lies in the use of neuropsychological and genetic data from FEP patients and their unaffected first-degree relatives. However, some limitations should also be acknowledged. First, the modest sample size of the study, especially when analysing subgroups, and the incomplete families with only sibling pairs limit the study of genetic transmission. In this regard, beyond larger samples future studies would also benefit from including both first-degree relatives of controls and affected and non-affected first-degree relatives of patients. Second, IQ estimation focuses on crystallised intelligence, and the results may not generalise to other types of intelligence such as fluid intelligence. Third, the inclusion of participants of European ancestry may limit the generalisation to diverse populations. Finally, potential biases may also arise from voluntary participation and the exclusion of relatives with a history of psychiatric diagnosis, which may result in a sample with preserved cognitive function. Further studies involving two or more people with psychosis in the same family may be relevant for studying populations at high risk of SCZ.

## Conclusions

Based on a family-based design in an FEP population, we confirmed that the polygenic risk for SCZ is increased in the probands, whereas the first-degree relatives score is intermediate between patients and controls. This validates the polygenic background as a discernible marker of genetic risk variation within families. Additionally, our results indicated that the genetic load for SCZ significantly predicts the deviation from the family-IQ, explaining that FEP patients underperformed in the IQ test compared to their relatives. The genetic risk for SCZ may modulate cognition by shaping developmental trajectories and making individuals more sensitive to environmental insults, therefore, preventing individuals from reaching the familial cognitive potential. Further research is needed to determine the potential contribution of genetic liability for intelligence to the unrealised cognitive potential of FEP patients.

## Supporting information

Murillo-García et al. supplementary material Murillo-García et al. supplementary material

## Supplementary material

The supplementary material for this article can be found at http://doi.org/10.1192/j.eurpsy.2024.24.
