# Supplementary material for: Studying the relationship between intelligence quotient and schizophrenia polygenic scores in a family design with first-episode psychosis population
Source: Eur Psychiatry. 2024 Mar 11;67(1):e31. doi: 10.1192/j.eurpsy.2024.24 (PMC11059248; doi:10.1192/j.eurpsy.2024.24)
Supplement: Murillo-García et al. supplementary material [file S0924933824000245sup001.docx]

**Supplementary material**


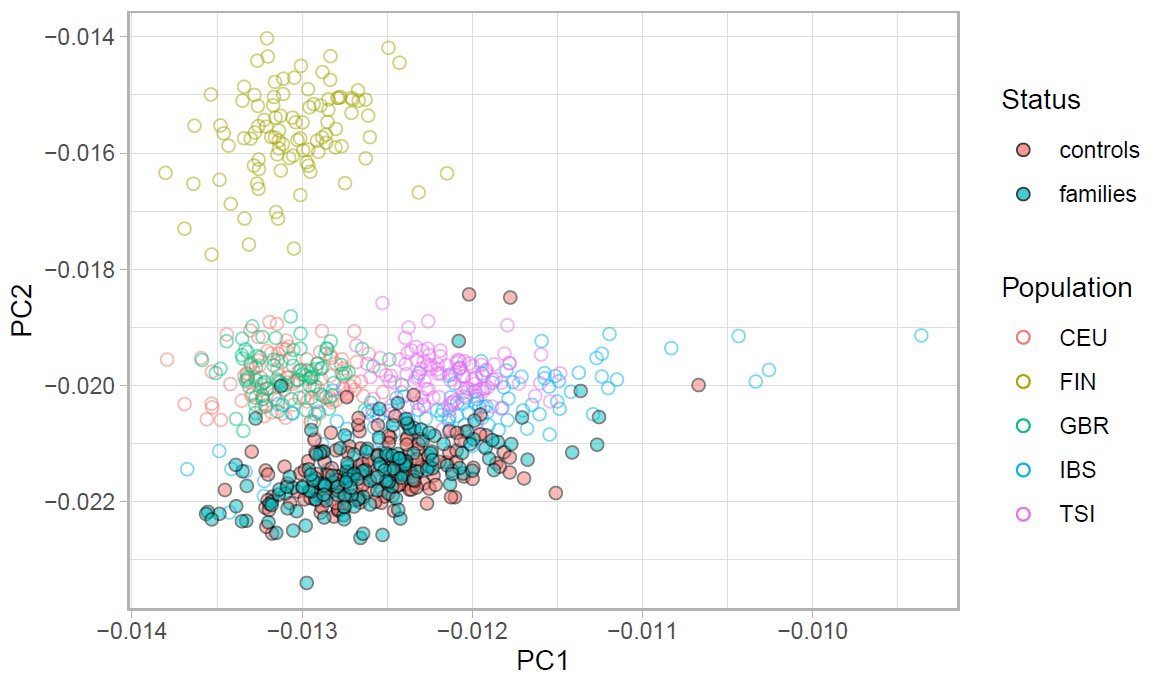


**Figure 1.** **Scatter plot of the homogeneity of the participants based on ancestry principal components.** The x-axis represents the first principal component (PC1), and the y-axis represents the second principal component (PC2). Based on 1000 Genomes Project European reference populations, the plot shows that our study participants (both families and controls) overlap with the Iberian population.

**Notes on the estimation of deviation from family-IQ**

Family-IQ was estimated in two different ways: 1) with all family members including patients (M= 109.25, SD= 9.32); 2) only unaffected relatives without patients (M= 105.13, SD= 8.90). Both measures are highly correlated (*r*= 0.83, *p*<0.001). Given the distribution of our sample, we performed the main analysis based on the family-IQ estimated on all participants.

The following Tables 1 and 2 show the results of the family-IQ as estimated on unaffected relatives only.

**Table 1. The predictive effect of PGS-SCZ on IQ deviation from unaffected relatives using linear mixed models.**

| Fixed effects | Beta coefficient standardized (SE) | *T* | *pFDR* |
| --- | --- | --- | --- |
| PGS-SCZ | -0.17(0.05) | -3.42 | <0.001 |
| Years of education | 0.18 (0.05) | 3.82 | <0.001 |
| Age | 0.39 (0.05) | 7.78 | <0.001 |
| Sex | 0.03 (0.05) | 0.69 | 0.487 |
| Overall model: *Wald*= 106.77, *p*<0.001, *R2*= 0.24 | | | |

**Table 2. The predictive effect of PGS-IQ on IQ deviation from unaffected relatives using linear mixed models.**

| Fixed effects | Beta coefficient standardized (SE) | *T* | *pFDR* |
| --- | --- | --- | --- |
| PGS-IQ | 0.10 (0.05) | 2.12 | 0.045 |
| Years of education | 0.19 (0.05) | 4.10 | <0.001 |
| Age | 0.43 (0.05) | 8.90 | <0.001 |
| Sex | 0.02 (0.05) | 0.500 | 0.617 |
| Overall model: *Wald*= 97.73, *p*<0.001, *R2*= 0.22 | | | |
